# Design and Validation of a Device Attached to a Conventional Bicycle to Measure the Three-Dimensional Forces Applied to a Pedal

**DOI:** 10.3390/s21134590

**Published:** 2021-07-04

**Authors:** Ezequiel Martín-Sosa, Víctor Chaves, Ignacio Alvarado, Juana Mayo, Joaquín Ojeda

**Affiliations:** 1 Departamento de Ingeniería Mecánica y Fabricación, Escuela Superior Técnica de Ingeniería, Universidad de Sevilla, 41092 Sevilla, Spain; chavesrv@us.es (V.C.); juana@us.es (J.M.); joaquinojeda@us.es (J.O.); 2 Departamento de Ingeniería de Sistemas y Automática, Escuela Superior Técnica de Ingeniería, Universidad de Sevilla, 41092 Sevilla, Spain; ialvarado@us.es

**Keywords:** cycling, 3D pedal forces, strain gages, lateral-medial force, tangential force, radial force, pedalling power, effectiveness index

## Abstract

Knowledge of the forces applied to the pedals during cycling is of great importance both from the point of view of improving sporting performance and medical analysis of injuries. The most common equipment for measuring pedal forces is usually limited to the study of forces in the sagittal plane. Equipment that measures three-dimensional forces tends to be bulky and to be incorporated into bicycles that are modified to accommodate it, which can cause the measurements taken to differ from those obtained in real pedalling conditions. This work presents a device for measuring the 3D forces applied to the pedal, attachable to a conventional bicycle and pedals, which does not alter the natural pedalling of cyclists. The equipment consists of four gauges located on the pedal axis and two on the crank, controlled by a microcontroller. Pedal forces measurements were made for six cyclists, with results similar to those shown in the literature. The correct estimation of the lateral-medial direction force is of great interest when evaluating a possible overload at the joints; it will also allow a comparison of the effectiveness index during pedalling, showing the role of this component in this index from a mechanical standpoint.

## 1. Introduction

Cycling has seen exponential growth in recent years. Therefore, interest has increased as regards the different parameters involved in the activity, such as the force exerted on the pedal. In the professional field, pedalling force is monitored to establish bicycle adjustments that enable maximum application of effective force (tangential to the crank), with minimum risk of injury [1]. As regards the clinical field, cycling is an excellent activity for the process of rehabilitation [2,3], in which it is essential to control the pedalling forces that the patient can exert during therapy sessions. Regarding recreational cycling, more and more users want to understand the characteristics of their pedalling to improve their technique. The movement during pedalling takes place mainly in the sagittal plane, which is why most studies focused on pedal forces have been devoted to analysing forces in this plane [4,5,6,7,8,9,10,11]. However, the analysis of the forces exerted in the three directions of space, including the force in the lateral-medial direction is convenient for an advanced study.

The quantification of lateral-medial force is important because it has a negative effect on pedalling performance by not contributing to crank movement. In addition, high values of lateral-medial force can generate reaction forces in the cyclist that lead to injuries, for example, to the knee [3]. There are several reports in the literature devoted to the study of three-dimensional forces on the pedal. Davis and Hull [12,13] developed a device to measure the three forces and three moments on the pedal. It consisted of 32 strain gauges embedded inside a modified pedal body and under the pedal axis. This equipment located on the pedal was of considerable size and only suitable for laboratory use. Nabinger et al. [14] used a platform attached to the pedal consisting of several deformable beams in which strain gauges were incorporated to measure the three forces and two moments on the pedal. Mornieux et al. [15] used a cycle ergometer mounted on a force platform to measure the three forces applied to the right and left pedals. The equipment was intended for laboratory use only. Alexander et al. [16] developed a pedal equipped with strain gauges and a potentiometer to measure three-dimensional forces on the pedal, usable both in the laboratory and outdoors. Lee et al. [17] also developed a pedal to measure three-dimensional forces on the pedal.

Recently, Dieltiens et al. [18] patented a low-cost pedal to measure these forces, based again on a strain gauge platform attached to the pedal axis. The force measurements obtained with all of these devices may differ somewhat from the forces produced under real pedalling conditions, due to commercial pedals not being used, but rather measuring platforms with a shape similar to that of a pedal. The platforms are relatively bulky in some of these devices, causing the distance from the foot to the pedal axe to be slightly different from that of a conventional pedal. In addition, these platforms, with a lot of instrumentation included, can be heavier than those of commercial pedals, which are currently very light. Therefore, it is foreseeable that the pedalling of cyclists will differ, at least slightly, with respect to that performed on a bicycle with the usual commercial pedals, and therefore it is foreseeable that the forces measured on the equipment will differ from those performed in real conditions with commercial pedals. To the best of our knowledge, currently there is no equipment for measuring three-dimensional pedal forces that can be used in the laboratory and outdoors and that can be fitted to a commercial bicycle and pedals without altering the rider’s natural pedalling in any way.

The aim of this research was the development of a device to measure the three-dimensional force on the pedal during the pedalling of a cyclist. The main design requirements were two: firstly, to develop a device that can be implemented on a conventional bicycle and pedal that does not alter the pedalling pattern of the cyclist; and secondly, to accurately measure the force in the lateral-medial direction because of its importance in the estimation of the reaction forces in the joints of the cyclist. Secondary objectives were the analysis of the pedalling effectiveness index and the study of the influence of the 3D character on the mechanical pedalling performance.

## 2. Materials and Methods

### 2.1. Measurement Equipment

Forces applied to the pedal measurements were carried out by installing strain gauges due to their flexibility to adapt to any type of pedal and their small size, following the line marked by the studies of Pigatto et al. [19], Bini et al. [20] or Balbinot et al. [21]. These projects tend to use a high number of gauges or bulky equipment that can affect the well-being of the subject during the measurements. To avoid this, one of the premises of this project was to use a reduced number of gauges, which significantly minimises the size of the measuring equipment, making it adaptable to any bicycle model.

**Figure 1:**
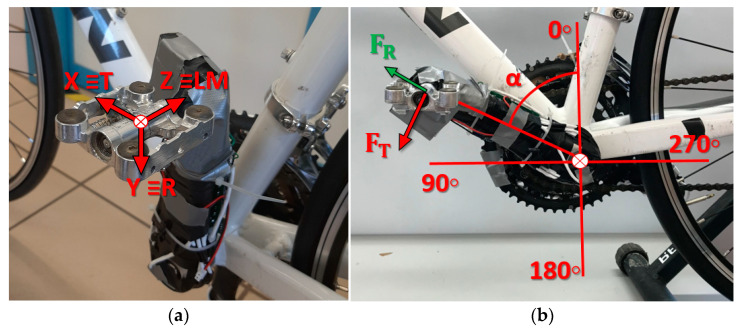
Measurement equipment details. (**a**) Coordinate origin and system. T: tangential direction. R: radial direction. LM: lateral-medial direction. (**b**) Crank rotation angle, α, with respect to a vertical line and forces components F_T_ and F_R_ apply to the pedal.

The first step for the pedal instrumentation was to choose the coordinate system with which to define the components of the force to be measured. This coordinate system can be seen in Figure 1a. A system of axes was defined in solidarity with the pedal axe and the crank as follows: *x* axis in the direction tangential to the path of the point where the crank meets the pedal axe; *y* axis in the direction of the crank axe; *z* axis in the direction of the pedal axe (Figure 1b). This definition of the coordinate system was made for the left pedal. A similar definition could be made for the right pedal, taking into account the plane of symmetry defined by the bicycle frame. For the definition of the forces the following nomenclature is used, the force applied in the *x* direction will be called tangential force, F_T_, to the trajectory of the crank end. The force applied in the *y* direction is defined as radial force, F_R_, following the direction of the crank axis. These two force components are located in the sagittal plane, parallel to the bicycle frame, Figure 1b. The force component in the *z* direction, direction of the pedal axis, is called lateral-medial force, F_LM_. According to these definitions, only the force F_T_ will produce a useful moment that rotates the crank. The other two forces, F_R_ and F_LM_, will not produce any useful moment on the crank.

**Figure 2:**
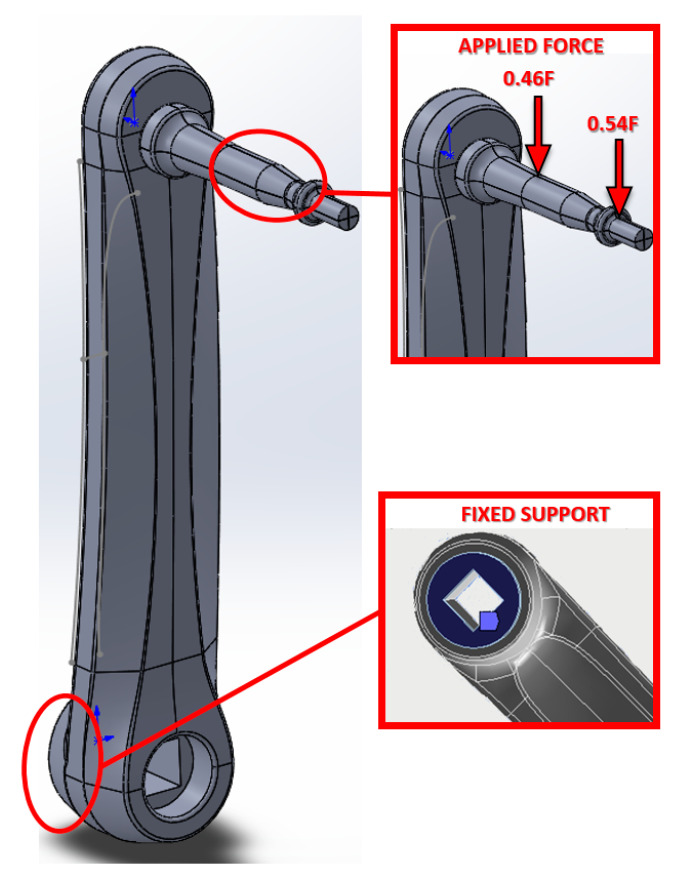
Finite element crank and pedal model. Applied force detail. Fixed Support contour boundary.

Theoretical calculations and finite element simulations were carried out to choose the optimum location for the gauges. The analyses were carried out in Ansys^®^ 19.2 for a conventional pedal and crank. The crank model was Triban 500 and the pedal model was WPD-M17C. SolidWorks^®^ 2018 was used to model the crank and pedal axis. The relative displacement between the pedal axis and the crank was prevented in the finite element model. As a boundary condition, the relative rotation of the crank in relation to the bottom bracket was prevented (see Figure 2). Each simulation consisted of applying a load of 98.1 N, 10 kg, in each of the defined directions. The applied force was assumed to be pointwise at the centre of the pedal. However, the transmission of this force to the pedal axis occurs via two bearings placed in the pedal axis. Therefore, in order to simplify the study of pedal axis and crank deformations, the pedal body was not included in the finite element study, although its transmitting effect of the force applied at the centre of the pedal was included. This behaviour was achieved by applying two point forces at the centre of each of the two bearings, distributed according to the geometry of the pedal and the position of the bearings, Figure 2. The two bearings were not exactly the same and were not equidistant from the point of application of the point force, the outer bearing being closer to the point of application than the inner bearing. Making a simple calculation considering the central point of the two bearings, the proportion of the applied point force F that was transmitted to the shaft by each bearing was obtained, being 0.54F in the outer bearing and 0.46F in the inner bearing. In any case, the way in which the force on the pedal was transmitted to the pedal axis was an intrinsic characteristic of the type of pedal and axis used, without affecting the general methodology undertaken in this work.

**Figure 3:**
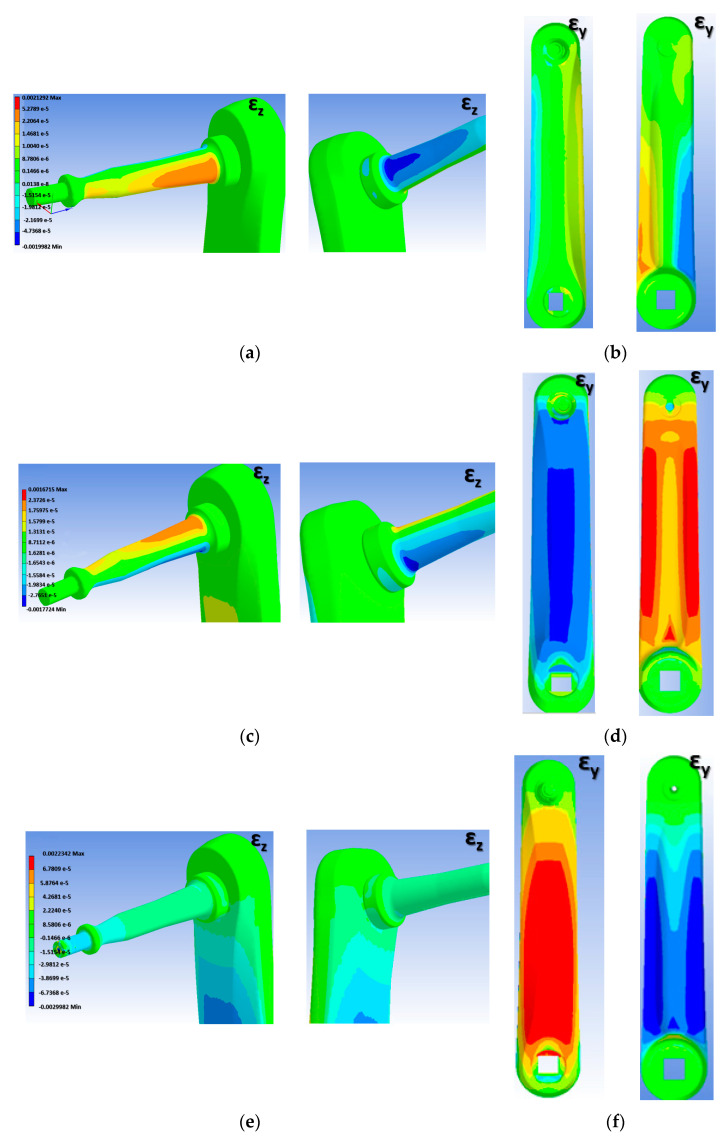
Pedal and crank strain mapping. (**a**) Strain in *z* direction (ε_z_) when F_T_ is applied. (**b**) Strain in *y* direction (ε_y_) when F_T_ is applied. (**c**) Strain in *z* direction (ε_z_) when F_R_ is applied. (**d**) Strain in *y* direction (ε_y_) when F_R_ is applied. (**e**) Strain in *z* direction (ε_z_) when F_LM_ is applied. (**f**) Strain in *y* direction (ε_y_) when F_LM_ is applied.

Figure 3 shows the strain map of the system under the three defined load cases. The analyses show how the F_T_ force produces bending of the pedal axis, generating high deformations, equal and of opposite sign, in the front and rear areas of the pedal axis, Figure 3a, according to the local reference system defined in Figure 2. The F_R_ force produces a bending of the pedal axis in the *y*-*z* plane, generating high deformations in the upper and lower areas of the axis, Figure 3c. Additionally, this state of loading causes a state of compression and bending in the crank, as shown in Figure 3d. The F_LM_ force produces a compression of the pedal axis, which generates much smaller strain than the bending of the pedal axis, Figure 3e. However, this F_LM_ force does produce bending of the crank, Figure 3f. Based on the results of the analysis and simulations, the most appropriate way to measure the forces in the tangential and radial direction was by placing gauges on the pedal axis positioned at 90°, coinciding with the areas of greatest strain obtained in the simulations. In order to accurately estimate the force in the lateral-medial direction, gauges were placed in the area of the crank closest to the embedding due to the high level of strain obtained in the simulations.

**Figure 4:**
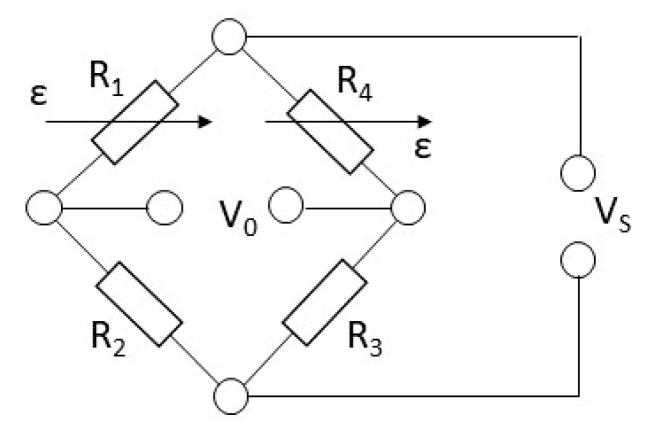
Wheatstone bridge circuit diagram. Half-bridge configuration. Vs: input voltage. Vo: output voltage. Ri: resistance i. ε: indicates which resistances represent strain gauges.

**Figure 5:**
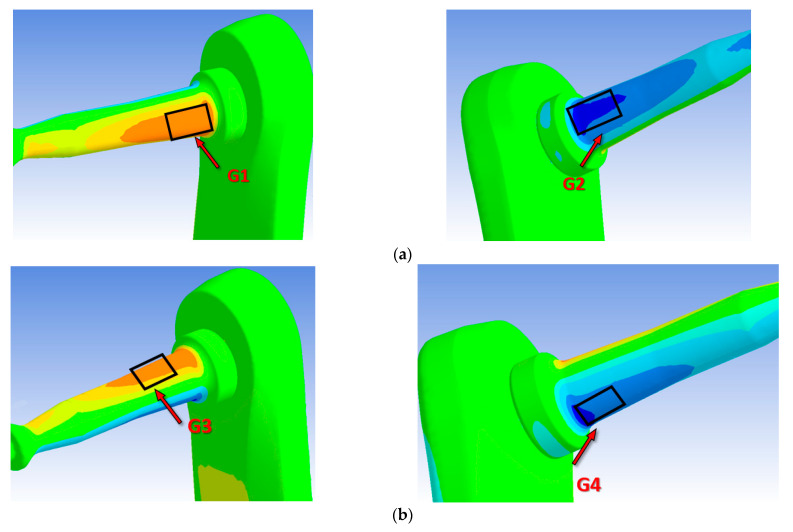

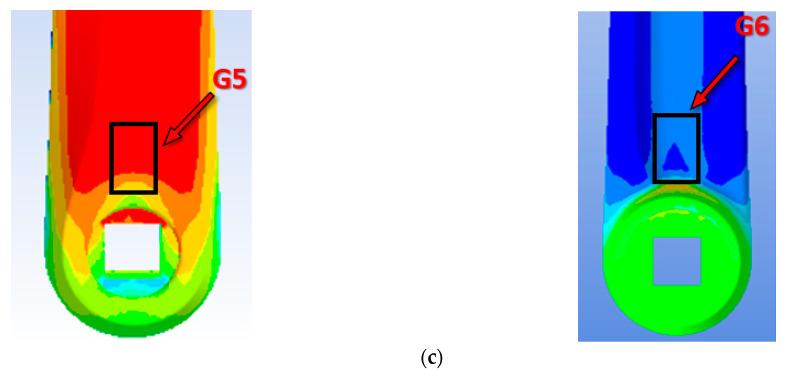
Gauges positioning in the different pedal areas. (**a**) Wheatstone bridge 1. (**b**) Wheatstone bridge 2. (**c**) Wheatstone bridge 3.

Two different models of gauges were used to ensure an adequate thermal compensation factor due to the pedal axis being made of steel and the crank of aluminium. For the axis, the CEA-06-062UW-350 model was used, and for the crank, the CEA-13-062UW-350 model was used. To obtain a signal from the gauges, they had to have been installed in a Wheatstone bridge circuit. In this type of circuit, Figure 4, one or more resistors in the circuit correspond to strain gauges placed on the element to be measured. When a certain electrical input voltage is applied, if the strain gauge is deformed, its resistance changes, and therefore the output voltage and an equation can be obtained that relates the deformation that occurs in the area where the strain gauge is placed and the ratio between the input and output voltage of the circuit. To improve the stability of the signal coming from the Wheatstone bridge, two gauges were installed in each area of interest, forming a Wheatstone bridge in a half-bridge configuration using a total of 6 gauges to form 3 Wheatstone bridges. Figure 5 shows the placement zones of the 6 gauges according to the level of deformations obtained in each of the analyses carried out.

**Figure 6:**
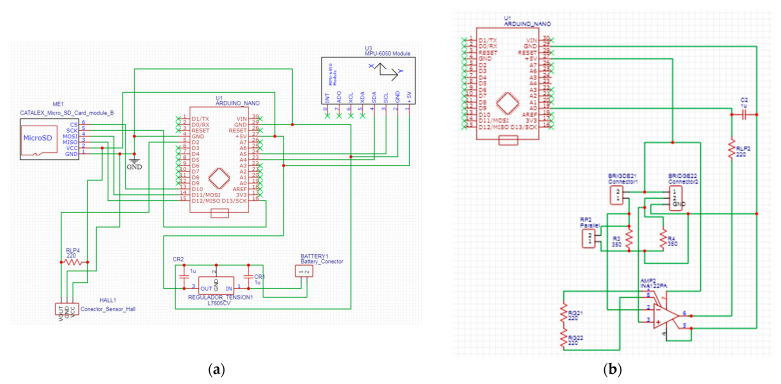
Diagram of the elements used in the PCB. (**a**) Power supply and data storage circuit with voltage regulator. (**b**) Half-Wheatstone bridge measurement circuit with low-pass filter.

Following the nomenclature shown in Figure 6, the Wheatstone bridge 1 is formed from gauges G1 and G2; this configuration of gauges makes this bridge measure mainly the tangential force component. Wheatstone bridge 2 is formed from gauges G3 and G4; the position and orientation of these gauges is used to measure the radial force component. Wheatstone bridge 3 is designed to measure the lateral-medial force component from gauges G5 and G6. In this configuration, the imbalance of both gauges will serve to measure one main force component. However, due to the geometry and the point of application of the load, the rest of the bridges may be activated to a greater or lesser extent.

Table 1 shows the values in micro-deformations provided by each gauge when a 10 kg weight is applied in the three directions: tangential, radial, and lateral-medial. The analysis of the results shown in Table 1 indicate that when the weight is loaded in the tangential direction, gauges G1 and G2 undergo the greatest deformation, Figure 3a, with practically equal values, but with the opposite sign due to the axis being subjected to bending. The rest of the gauges showed values close to zero. These results are coherent since for this state of stresses the areas where the gauges are located for bridges 2 and 3 should hardly suffer any deformation. In any case, the values shown in Table 1 are an average of the deformations obtained in the concurrent nodes in the area where the gauge is placed. The signal from gauges G1 and G2 give measurement values close to zero when load is applied in the radial direction, Figure 3c. In this case the area where gauges G3 and G4 are installed will be under bending, as will the area where gauges G5 and G6 are located, Figure 3d. Therefore, the signal of both bridges will be significant The results shown in Table 1 show for this case how the strain values obtained are practically the same and of opposite sign in the gauges of these two bridges. Finally, when the load is exerted in the lateral-medial direction, Figure 3f, gauges G5 and G6 will be under bending stresses, which is the reason why the deformations obtained in the areas where these gauges are placed are greater. Likewise, it can be seen that the deformations are the same, but of opposite sign. The deformations measured in the areas where gauges G1 to G4 are placed are very small because the pedal axis is axially stressed and the stiffness is very high, Figure 3e. However, these results are consistent because the deformations in the four gauges of the pedal axis are equal and negative due to the axis being subjected to compressive stress.

In addition to estimating the forces applied to the pedal by means of Wheatstone bridges, the pedal instrumentation also aimed to analyse the instantaneous angular velocity of the crank, the position of the crank and the number of turns the crank makes during each trial. For this purpose, an inertia measurement unit (IMU), MPU6050 model, and a Hall effect sensor were included; this sensor resets the accumulated error produced by the IMU gyroscopes necessary for the calculation of the crank position by integrating the angular velocity. In order to be able to store all the generated data, a microSD module was also provided, Figure 6a. All these devices are controlled by an Arduino Nano microcontroller with an ATMega 328P processor through a 10-bit analogue-to-digital converter (ADC), Figure 6b.

Due to the signals obtained when the Wheatstone bridges are unbalanced and are of the order of millivolts, imperceptible to the ADC of the Arduino, it is necessary to amplify them. These signals are amplified by an operational amplifier, model INA 122. The gain value is in the order of 500 *V*/*V* and is set by the resistors, RG_21_ and RG_22_. The gain value must ensure that the forces applied by an average cyclist do not exceed the 0 to 5 V limits of the ADC of the Arduino inputs because the Arduino is not able to recognise negative signals. To ensure that the signals coming from the amplifier are positive, the Wheatstone bridges were unbalanced to make a voltage of 2.5 V equivalent to a 0 N force. The unbalancing of the bridges was carried out by placing a fixed resistor in parallel, RP_2_, to one of the resistors that close the bridge (Figure 6b). The amplified signal presented high-frequency noise; for this reason, a low-pass filter was installed at the output of the amplification stage of each Wheatstone bridge, consisting of a 100 µF capacitor, C_1_, and a 220 Ω resistor, RLP_1_, to eliminate this noise (Figure 6a).

**Figure 7:**
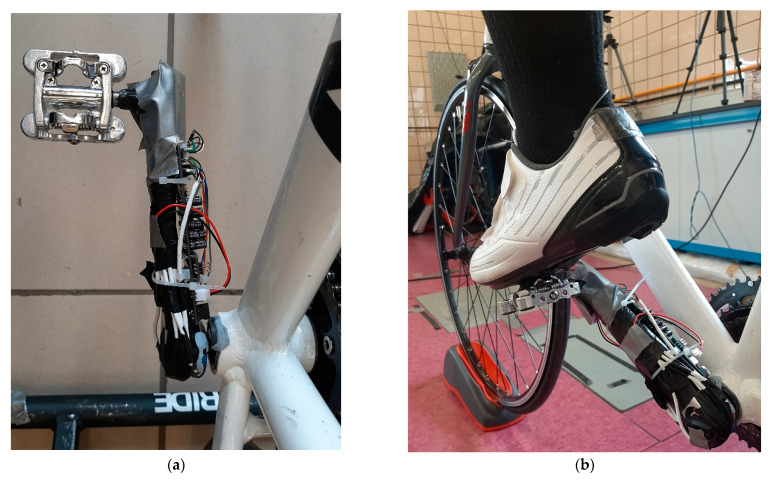
Measurement equipment located on the bike. (**a**) Location. (**b**) Pedalling test.

A 9 V battery was used to power all the sensors, Wheatstone bridges, SD module, microcontroller and auxiliary electronics. Due to the way in which the battery delivers the voltage, it is usual to see oscillations and drifts in the signals read by the microcontroller. To avoid this undesired behaviour, a voltage regulator was installed, model L7805CV, Figure 6a. This electronic element maintains the constant value of the power supply voltage, giving all the measurement signals greater robustness and stability in the measurements. To ensure that the assembly of all these components does not affect the well-being of the participant during pedalling and does not take up a large amount of space, a printed circuit board (PCB) of dimensions 100 mm × 45 mm was developed, on which all the electronic components were soldered. This PCB was placed on the crank internal side and had no impact on the pedalling of the participant, Figure 7. Both the circuit diagram and the PCB design were carried out using the EasyEDA software.

An important aspect in estimating the value of the forces is to ensure thermal compensation in the Wheatstone bridges. Firstly, the selected gauge models were self-temperature-compensated for use on structural materials with specific thermal expansion. Secondly, each time a measurement was taken, the system was switched on and left to operate without any force applied for 5 min. After this time, the established value of each Wheatstone bridge was recorded and considered as the value corresponding to zero deformation. Finally, the passage of electricity through the circuits forming the Wheatstone bridge caused the gauges to heat up and their temperature to increase, which stabilised after a few seconds. However, in this work, the gauges used were 350 ohms, which is a relatively high resistance value and minimises the power consumed by the gauge and, therefore, the temperature rise. In any case, given the characteristics of the gauges used the effect of temperature on the response of the gauges was assumed to be negligible in the range of 10 to 40 °C. Thus, the device could be used outdoors in a wide range of climatic conditions.

Once the different measurements were taken and the data were saved on the SD card, they had a range of values between 0 and 1023, because the Arduino ADC digitises the input signal (volts) and transforms it into bits. These files were processed in Matlab^®^ 2017, where the first thing that was done was to compensate for the initial unbalance of the bridges, with the average of the values saved in the first 75 s of each session. To further reduce the noise that was present in the signals, a digital low-pass filter was applied to the data collected from the pedal. The filter expression is as follows:

(1) $$S_{i}^{fil}=\alpha \cdot S_{i-1}^{fil}+\left(1-\alpha \right)\cdot S_{i}$$

where $S_{i}^{fil}$ is the signal of each bridge filtered at instant *i*, $S_{i-1}^{fil}$ is the signal filtered just the previous instant and $S_{i}$ is the unfiltered signal at instant *i*. The values of the parameter α are in the range (0.5, 0.95).

### 2.2. Experimental Force Determination

In order to convert the bits from the Arduino into force values, it was necessary to apply conversion factors to each pedal separately. These factors were calculated from the following system of equations:

(2) $$f=M\cdot e_{g}$$

where **f** is the column vector formed by the three components of the force, **M** the 3 × 3 matrix that integrates the bit-Newton conversion factors and **e_g_** the column vector with the Arduino data for the different bridges. This way of estimating the forces has already been carried out by other studies [22].

The fact that both the pedal axis and the crank were made of commercial steel and aluminium alloys made it possible to assume a linear and isotropic elastic behaviour of both elements. This hypothesis, combined with the assumption that the electronic device has a linear response, allowed the values of the applied forces to be determined from the values provided by the microcontroller, **e_g_**, and the **M** matrix.

In order to obtain the conversion factors that form the **M** matrix, 110 calibration tests were performed on the pedal. These tests consisted of the application of a known force in each of the three directions, tangential, radial and lateral-medial. The direction of force application and crank angle were ensured to be correct in all tests by the use of a goniometer. Subsequently, a correlation analysis was performed to validate the assumed linearity hypothesis.

From an electronic point of view, there is no cross-sensitivity in the gauges because the only common point between the measuring circuits is the power supply, which is controlled by the L7805CV voltage regulator. Therefore, as long as the value of 800 mA is not exceeded, the voltage at that point is guaranteed to be 5 V, regardless of the load.

For each test, data were collected from the three Wheatstone bridges, and three systems of equations were obtained, one per axis direction. For this case the matrix **M** was the unknown. Coupling the three systems and operating so that the elements of the matrix **M** were positioned in a column vector **m**, a system of matrix equations of dimension 9 × 9 was obtained.

The system was of the following form:

(3) $$f_{cal}=B\cdot m$$

where **f_cal_** represents the 9 × 1 column vector that integrates the three components of the force applied in the three calibration tests. **B** is a 9 × 9 matrix that collects the response of the three gauges for the three tests, and **m** is a column vector, 9 × 1, formed by the 9 conversion factors that relate the response of the gauges to the applied force.

### 2.3. Calibration Results Assessment

The deviation between the applied forces and the estimated forces was quantified using the root-mean-square error normalised to the modulus of the applied weight.

(4) $$NRMSEM_{jp}^{}=\frac{\sqrt{\frac{1}{n}\sum_{i=1}^{n}{\left(F_{jpi}^{o}-F_{jpi}^{a}\right)}^{2}}}{\left|{\vec{F}}_{p}^{a}\right|}\;\cdot 100$$

where $NRMSEM_{jp}^{s}$ is the normalised root-mean-square error for the $M_{jp}^{}$ matrix on the *j* axis direction for a calibration test *p*. The number of instants at which the calibration load was applied is denoted by *n*, *F_o_* is the calculated force value for the *j* axis, *F_a_* the theoretical force applied on the *j* axis direction and $\left|\vec{F_{a}}\right|$ the modulus of the force vector due to the calibrated weight. Thus, each matrix $M_{jp}^{}$ would yield three errors (one per axis direction) for each test (regardless of the axis direction on which the load will be applied). Additionally, for each test, the global average error was also calculated, using the following expression:

(5) $$NRMSEM_{p}=\frac{NRMSEM_{Xp}+NRMSEM_{Yp}+NRMSEM_{Zp}}{3}\;$$

In addition to the normalised mean-square-error of each axis direction for each calibration test, Equation (4), and the overall average error of each calibration test, Equation (5), the average error of each axis direction for the 110 calibration tests performed was calculated using Equation (6). The overall average error for all calibration tests was obtained using Equation (7).

(6) $$NRMSEM_{j}=\overset{110}{\underset{p=1}{\sum }}NRMSEM_{jp}\;$$

(7) $$NRMSEM_{global}=\overset{110}{\underset{p=1}{\sum }}NRMSEM_{p}\;$$

### 2.4. Test Conditions

For the validation of the dynamic tests, the pedalling of a series of participants was analysed. The tests were carried out on the Triban 500 commercial bicycle, which was mounted on an Elite Roller training roller. The bicycle was equipped with clipless pedals, and the participants wore shoes with cleats. Saddle height was calculated using the methodology developed by Holmes et al. [23]. The pedalling cadence was set to 60 rpm, controlled by an acoustic signal. The pedalling power was set to 150 W and was guaranteed by the set cadence, the gear ratio setting and the resistance of the training roller. The hands were placed at the top position of the handlebars.

Before the participant got on the bike, the measurement of the equipment was started, in order to record the value used as 0 for the Arduino inputs. This process lasted approximately 75 s. Once this process was completed, the dynamic tests were carried out under the established test conditions. The duration of each test was 10–15 min, using the first 2 min for a light warm-up and adaptation to the established conditions. Once the warm-up and adaptation period was completed, the forces applied to the pedals, the power exerted on the pedal and the cadence of the test were analysed.

### 2.5. Effectiveness Index Analysis

One way to measure the performance of a cyclist is through the study of the effectiveness index. This index helps to identify the percentage of force applied to the pedals that produces a useful moment in the crank. For the estimation of this index, the literature shows many ways to calculate it; the most used is the one proposed by Bini et al. [20], Equation (8).

This index provides a scalar value; moreover, it will be obtained using only forces contained in the 2D plane.

(8) $$EI_{2D}^{}=\frac{\int_{0}^{tf}F_{T}\;dt}{\int_{0}^{tf}\left|\vec{F_{2D}^{}}\right|dt}$$

where $F_{T}$ is the useful force, tangential force, performed by the participant at instant *i* and $\left|\vec{F_{2D}}\right|$ is the modulus of the vector of the applied force during pedalling in the plane parallel to the bicycle frame.

This research aimed to compare the effect of the lateral-medial force component. Therefore, the effectiveness index was calculated in the same way as Bini [20] but including the lateral-medial component, Equation (9):

(9) $$EI_{3D}^{}=\frac{\int_{0}^{tf}F_{T}\;dt}{\int_{0}^{tf}\left|\vec{F_{3D}^{}}\right|dt}$$

For this equation $\left|\vec{F_{3D}}\right|$ will be the module of the three-dimensional vector of the force applied to the pedal.

It was also of interest in this study to analyse the temporal evolution of the effectiveness index throughout a pedalling cycle, for which Equations (10) and (11) were applied.

(10) $$EIE_{2D}^{i}=\frac{F_{T}^{i}}{\left|\vec{F_{2D}^{i}}\right|}$$

(11) $$EIE_{3D}^{i}=\frac{F_{T}^{i}}{\left|\vec{F_{3D}^{i}}\right|}\;$$

where $F_{T}$ is the effective force performed by the participant at instant *I*, and $\left|\vec{F_{2D}^{i}}\right|$ is the module of the vector of the applied force during pedalling in the plane parallel to the bicycle frame at instant *i*. In addition, $\left|\vec{F_{3D}^{i}}\right|$ is the module of the three-dimensional vector of the force applied on the pedal at instant *i*.

To estimate the influence of the lateral-medial force on the average effectiveness index and on its temporal evolution, the relative error was calculated as shown in Equations (12) and (13).

(12) $$Error_{EI}=\frac{EI_{2D}^{}-EI_{3D}^{}}{EI_{3D}^{i}}*100\;$$

(13) $$Error_{EIE^{i}}=\frac{EIE_{2D}^{i}-EIE_{3D}^{i}}{EIE_{3D}^{i}}*100\;$$

## 3. Results

The results obtained during the work on the left pedal and the left crank are shown below. Firstly, the results relating to the calibration process of the device are grouped together. Secondly, the results related to the pedalling tests carried out in laboratory conditions are shown.

### 3.1. Device Calibration

The value of the components of the vector **m** as a function of the weights applied for each calibration is shown in Table 2. Matrix **M_1_** is defined from the calibration factors obtained by applying a load of 5 kg, 49.05 N, to the pedal. The rest of the matrices, **M_2_**, **M_3_**, **M_4_**, **M_5_**, **M_6_** and **M_7_**, are defined from calibration factors using weights with values representative of the forces applied during pedalling. In this case, weights were taken with the following values: 7.5, 10, 13, 15, 20 and 30 kg.

**Figure 8:**
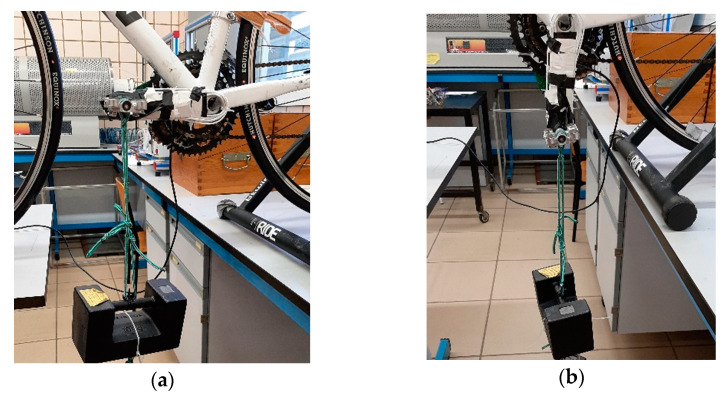

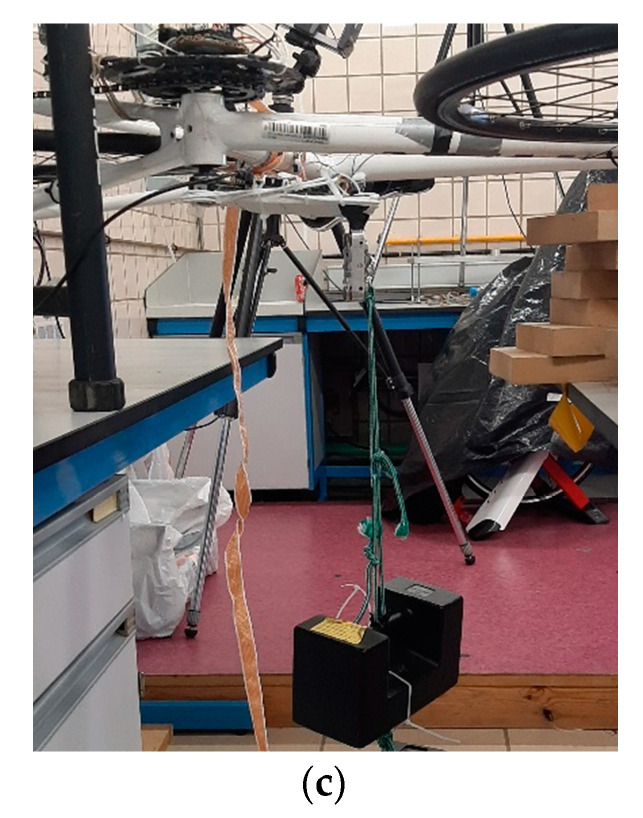
Pedal calibration procedure. (**a**) Tangential direction calibration mounting. (**b**) Radial direction calibration mounting. (**c**) Lateral-medial direction calibration mounting.

This table shows that for the different matrices, the value of the components of the vector **m** hardly varies due to the linear behaviour of the Wheatstone bridges as the load increases. The greatest variations are detected in the value of m_12_ and m_31_. For m_12_, the variations were due to the impossibility of systematically replicating the position of the crank for different weights for the tests in the tangential direction, Figure 8a. The second factor, m_31_, is made up of values in the order of hundredths, so any small variation in the calibration process could cause significant variations in this factor.

**Figure 9:**
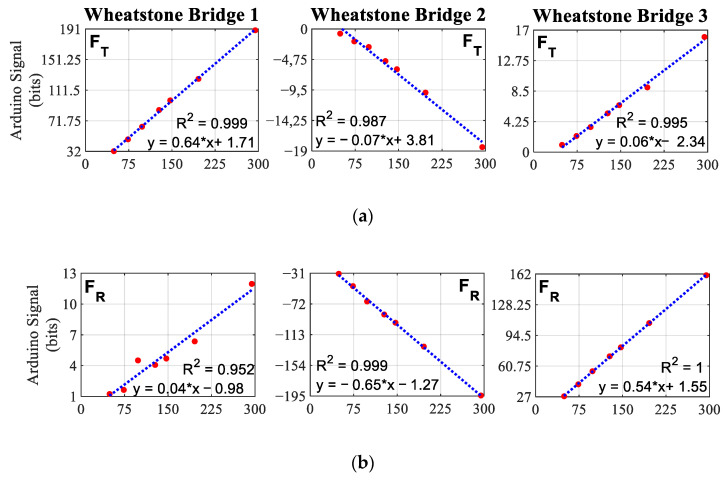

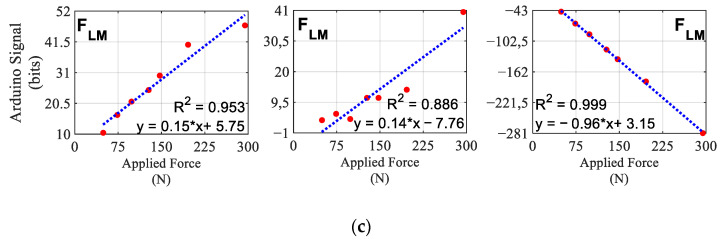
Regression lines of the signal of each Wheatstone bridge for different loads. (**a**) Results obtained when applying loads in the tangential direction. (**b**) Results obtained when applying loads in radial direction. (**c**) Results obtained when applying loads in lateral-medial direction.

The hypothesis of the linear behaviour of the measuring device is supported by the regression lines and the value of the correlation coefficient *R*^2^ shown in Figure 9. This figure shows the regression lines of the signal obtained in each bridge as a function of the load applied in each of the three directions of space. In this figure, when the tangential direction test is carried out, Figure 9a, Wheatstone bridge 1, which is the bridge responsible for measuring forces in that direction, shows an *R*^2^ of 0.999 and a maximum value in bits of 191. For that same test, the rest of the signals reach values of less than ±20 bits, close to 10% in relation to the signal measured by the main bridge in that direction. The value of *R^2^* for these two bridges is 0.987 for bridge 2 and 0.995 for bridge 3, showing a high linearity in their behaviour, although lower than that of bridge 1.

Radial direction calibrations, Figure 9b, show a similar behaviour. For this calibration, bridge 2 presents a maximum signal of −195 bits and bridge 3 of 162 bits; in contrast bridge 1, measures a maximum signal of 12 bits, being 6% of the maximum value of bridge 2. This behaviour is because a force applied in the radial direction generates a bending movement in both the pedal axis and the crank. The linearity in the signal with respect to the applied weight presented for this test is *R*^2^ = 0.952 for bridge 1, *R*^2^ = 0.999 for bridge 2 and *R*^2^ = 1 for bridge 3.

Figure 9c presents the correlation coefficient and regression lines of the 3 bridges for the calibration in the lateral-medial direction. For this direction, the linearity of the signals is somewhat lower and the noise of the bridges 1 and 2 somewhat higher compared with the rest of the directions. In this case the maximum value of the bridge 3 is −281 bits, and the value of the other bridges is 52 for the bridge 1, 18% of the value of the bridge 3 and 41 for the bridge 2, 14%. The linearity of the signals of bridge 3 presents a value of *R*^2^ = 0.999 and bridge 1 *R*^2^ = 0.953. For bridge 2, an *R*^2^ = 0.886 is obtained, the lowest of all calibrations.

Due to the validation of the hypothesis of the linear character of the measuring device, demonstrated in both Table 2 and Figure 9, the decision was taken to work with a single transformation matrix. The *m_ij_* factors of this matrix were obtained from the average of the corresponding factors of the seven conversion matrices shown in Table 2. The components of the resulting matrix, **M_mean_**, are shown in Table 3. If the values of the main diagonal of this matrix are compared with the values of the diagonal obtained for the different matrix, Table 2, they hardly differ by 4% with respect to the same **M_mean_** components. If the rest of the components are compared, the differences with respect to the value shown in **M_mean_** do not exceed 15%, with the exception of the m_21_ component. Since the value of this component for **M_mean_** is of the order of one hundredth, any variation, no matter how small, can cause deviations close to 30% of the value obtained in **M_mean_**.

Table 4 shows the root-mean-square error for each axis direction for the 110 calibration tests performed. The table shows that the values do not exceed 3.5%; to the best of our knowledge, these errors are lower than those obtained by other studies dedicated to the estimation of the force applied to the pedals [13,14,15,24].

**Figure 10:**
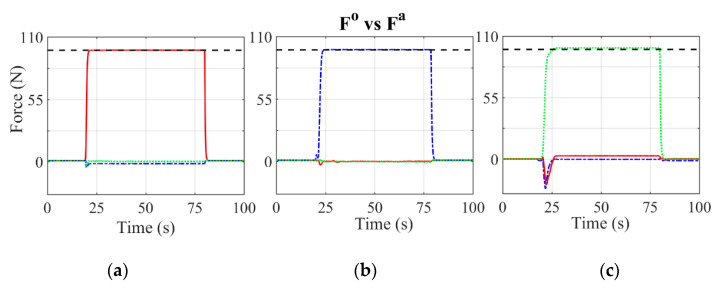
Measured forces (**F^0^**) in the tangential, radial and lateral-medial directions for the 10 kg calibration weight. **F^a^** is the calibration weight in N. (**a**) Calibration weight applied in tangential direction. (**b**) Calibration weight applied in radial direction. (**c**) Calibration weight applied in lateral-medial direction. Red solid line: tangential force. Blue dashed dotted line: radial force. Green dotted line: lateral-medial force. Black dashed line: calibration weight.

Figure 10 shows visually the behaviour of the evolution of the three force components for tests with a weight of 10 kg applied in each direction of the space, and Table 5 shows the global mean-square error of these tests. The value of the applied force is represented by a dotted black line. This type of test is used to determine the accuracy of the **M_mean_** matrix. The time evolution of the force components shows that for all cases, when no force is applied, the value of the three components is zero. When a force is applied in one of the space directions, the bridge signal responsible for measuring that component is the one that is activated approaching the theoretical force value; the rest of the components remain close to a zero or residual value. In Figure 10, some small peaks can be seen at the beginning of the force measurement; this behaviour is due to the instant of application of the weight, and this effect becomes significant for Figure 10c.

**Figure 11:**
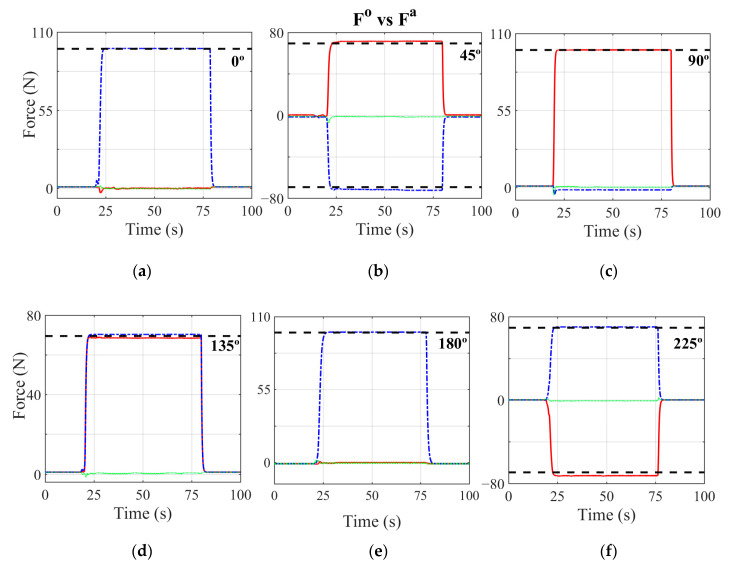

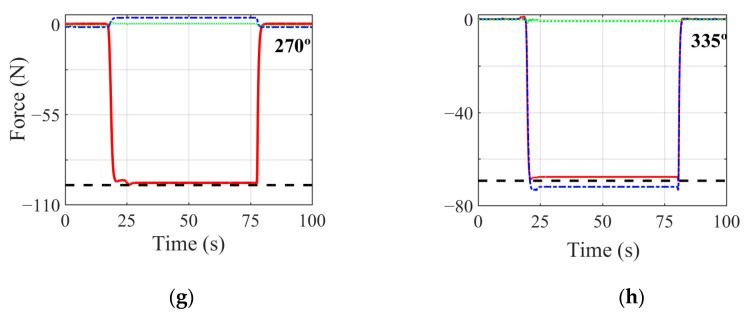
Measured forces (**F^0^**) in the tangential, radial and lateral-medial directions for the 10 kg calibration weight along different positions of the crank. **F^a^** is the theoretical weight in N. (**a**) Weight applied when the crank is at 0°. (**b**) Crank is at 45°. (**c**) Crank is at 90°. (**d**) Crank is at 135°. (**e**) Crank at bottom dead centre, 180°. (**f**) Crank is at 225°. (**g**) Crank is at 270°. (**h**) Crank positioned at 335°. Red solid line: tangential force. Blue dashed dotted line: radial force. Green dotted line: lateral-medial Force. Black dashed line: calibration weight.

Tests were carried out by applying a weight of 10 kg in nine different positions of the pedalling cycle, spaced 45° from each other to better understand the behaviour of the equipment at those points where calibration was not carried out. The quadratic errors of these tests are shown in Table 6, and the behaviour of each component during these tests is shown in Figure 11. Table 6 shows errors that do not exceed 4.5%. The largest value of this error is found in the radial component of the force when the force is applied at an angle of 270°. The lateral-medial component for all the tests has a value of 0 N, obtaining errors for this component close to 1%. Figure 11 shows how, depending on the position of the crank, the force components change direction. For those intermediate values of the crank, such as 45°, 135°, 225° and 315°, the device obtains very similar values between the components that are in the sagittal plane. This figure also shows how the lateral-medial component presents null values for all the tests carried out. The results obtained show that for all the tests carried out, the value of each component measured is very similar to the theoretical component.

### 3.2. Device Results in Pedalling Conditions

The pedalling of a total of six participants, adults and amateurs, with a mean age of 28 ± 7.61 years and a mean height of 1.76 ± 0.07 m were analysed; all of them participated voluntarily. None of the participants studied had any underlying pathologies. All the measurements were taken in a room in the laboratory of the Department of Mechanical Engineering and Manufacturing of the University of Seville. The room was air-conditioned, and temperature stability could be guaranteed in a range between 15 °C and 25 °C, which is within the correct operating range previously assumed.

**Figure 12:**
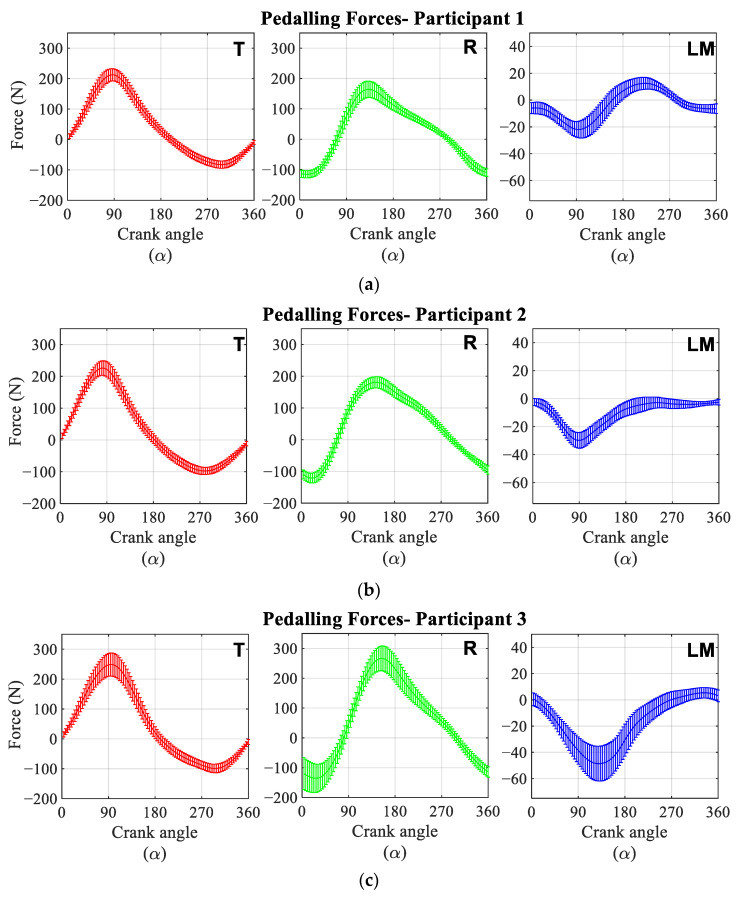

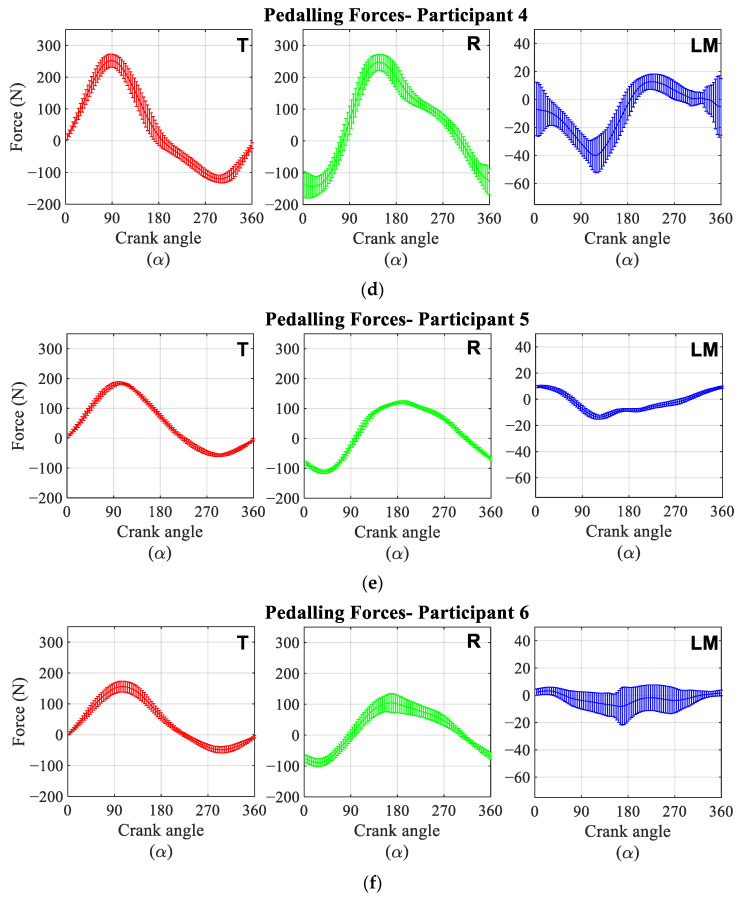
Mean pedalling forces time evolution applied to the pedal for each participant. Red: Tangential force (T). Green: Radial force (R). Blue: Lateral-Medial force (LM). (**a**) Participant 1. (**b**) Participant 2. (**c**) Participant 3. (**d**) Participant 4. (**e**) Participant 5. (**f**) Participant 6.

Figure 12 shows the average and standard deviation of each forces component during the pedal cycle of the six analysed participants. The graphs were normalised to show the crank angle, and the origin of the cycle was chosen at the top dead centre of the crank. All the participants exerted zero effective force, F_T_, at the beginning of the cycle. This force component reached its maximum value when the crank reached a position of 90° with respect to the vertical. It returned to zero at bottom dead centre and reached its minimum value when the crank was at 270°. Most of the participants exerted a maximum effective force of about 250 N, with the exception of participants 5 and 6, who exerted about 200 N and 150 N, respectively. The same tendency existed for the minimum force; these participants exerted a force close to −50 N while the rest of the participants applied a minimum effective force of −100 N. The radial force started the pedalling cycle near its minimum value and reached its maximum near the bottom dead centre. This component became zero when the crank was located near the 90° and 270° position. This component showed a greater difference between participants. Participants 1 and 2 exerted a maximum force limited between 150 and 200 N and a minimum force close to −150 N. Participants 3 and 4 were the ones who exerted the highest F_R_, reaching values close to 250 N. For these participants, the minimum value of the force is similar to the two previous participants, −150 N. Participants 5 and 6, as with the previous component, were those who exerted the least force for this component, with values close to 100 N for the maximum and −100 N for the minimum.

The lateral-medial force component, F_LM_, is the force exerted in the direction of the pedal axis. This component reached its minimum value when the crank was positioned close to 90°, not exceeding −50 N in any of the participants studied. After this position of the crank, this component tended to approach positive force values of no more than 15 N. Participant 3 was the one that reached the greatest negative force, −50 N, and participants 1 and 4 the ones that exerted the greatest forces, 15 N.

**Figure 13:**
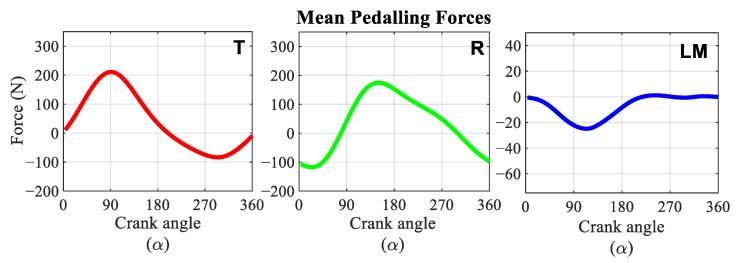
Mean pedalling forces time evolution applied to the pedal. Red: tangential force (T). Green: radial force (R). Blue: lateral-medial force (LM).

Figure 13 shows the average of the temporal evolution of the three components of the force during the pedalling cycle for all the analysed participants. For this case, a maximum F_T_ of 200 N and a minimum of −85 N were obtained. The radial force component applied to the pedal had a maximum value of 160 N and a minimum value close to −135 N. The average of the lateral-medial component barely reached positive force values and had a minimum value close to −25 N.

Table 7 shows the values of the temporal evolution of the effectiveness index for those moments in which it becomes maximum and minimum, which correspond to a crank position of 90° and 270°, respectively. Table 7 reflects that the values of the index calculated using a three-dimensional force are lower than index calculated using only two-dimensional forces, due to the modulus of the first one being slightly greater, but the differences found are in most cases of the order of the hundredth. If the maximum and minimum EIE value is compared in absolute value for each participant, all of them show a higher EIE value when the crank is at 90°, for both 2D and 3D. Table 8 shows the values obtained by applying the equations proposed by Bini [20] for both a 2D and a 3D study. In both tables a relative error is added, which serves to compare and quantify the effect of analysing the effectiveness index with or without the lateral-medial force direction.

**Figure 14:**
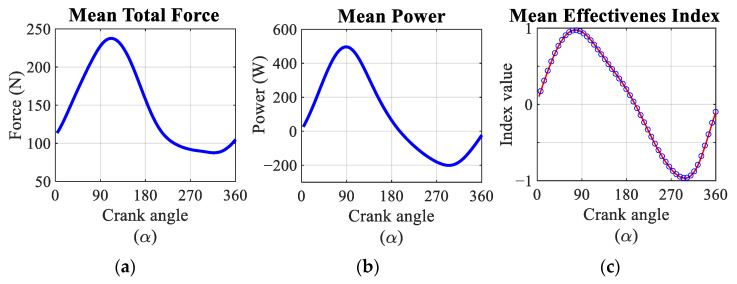
(**a**) Mean total force calculated as the result of F_T_ and F_R_. (**b**) Mean effective power time evolution applied to the pedal. (**c**) Comparison between the average of EIE_2D_ and EIE_3D_. Red dot and dash line: EIE_2D_. Blue circles: EIE_3D_.

The average of the evolution of the total force, the power and the effectiveness indices of the participants analysed during the pedalling cycle are shown in Figure 14. Total force reaches its maximum value, 230 N, when the crank is close to 90°, while its minimum value, 85 N, is reached when the crank is close to 330°. The average of the power reaches a maximum value close to 450 W. The minimum power reaches a value of −200 W.

## 4. Discussion

### 4.1. Device Calibration

The results obtained in the calibration of the device show the validity of the linearity hypothesis assumed in this study, having obtained correlation coefficients very close to unity. It is worth highlighting some comments on the analysis of these regression lines.

Firstly, the signals from the bridges 1, 2 and 3 have an *R*^2^ almost equal to unity when measuring loads in the tangential, radial and lateral-medial directions, respectively. The correlation coefficients of each bridge when applying loads in the other two directions are somewhat lower. However, the signals recorded in these cases are smaller than the diagonal terms of the calibration matrix. Therefore, errors made in the assumption of linearity have a practically negligible effect on the final force measurement.

Secondly, the linearity hypothesis assumed implies that the regression lines must pass through the origin, proving that in all cases the equations have a non-zero ordinate at the origin. The most significant cases are those obtained for bridges 1 and 2 at a lateral-medial load with an ordinate at the origin of 5.7 and 7.7 bits, respectively. These results can be explained by several reasons. The most important is that a strain gauge measures deformations not at a point but rather over an area of the surface of the object to be measured. Therefore, for a general loading state that should theoretically produce zero strain at a point, the strain gauge will register signals of the order of a few micro deformations or, in this case, bits. This result is in agreement with the deformation values shown in Table 1, where the microdeformations recorded correspond to the average of the deformations measured at the concurrent nodes within the strain gauge area. Additionally, errors due to deviations in the correct positioning of the strain gauge or in the application of the load are present.

These small deviations from the expected theoretical behaviour have not introduced a significant error in the results provided by the device, as could be verified. Firstly, the errors obtained with the calibration loads were less than 3.5%. Secondly, the measurements taken with the crank positioned at different angles of the pedalling cycle showed errors of less than 3%. In some exceptional cases where the crank was in the fourth quadrant, errors between 3% and 5% occurred. This increase in error was mainly due to the difficulty in properly positioning the calibration weight due to the roller support on the rear wheel of the bicycle. However, as mentioned above, these errors did not exceed 5% in any case.

The case of the lateral-medial direction calibration is particularly critical, as previously mentioned. According to the results obtained in the finite element model, the ratio between the deformations obtained in bridges 1 and 2 under axial and bending forces has a value of 4, with the deformations in the gauges of bridges 1 and 2 being of the order of 5 microdeformations under a force applied in the lateral-medial direction and 20 microdeformations under a force applied in the radial or tangential directions. However, when analysing these ratios on the experimental values obtained, certain differences can be observed. Specifically, in bridge 1 the ratio is approximately 3.07, and in the bridge 2 it is approximately 4.67. These differences may be due to several factors. Firstly, errors may be due to small misalignments of the gauges with respect to the lines of action of the calibration loads, as mentioned above. Secondly, errors may be due to noise in the measuring equipment. However, it is understood that these errors are very small. The main source of error may be due to the way the load is applied in the lateral-medial direction. Given the methodology employed, in which a weight is attached by means of a rope to the pedal axis, the load is applied eccentrically to the pedal axis. This can lead to a state of stresses and deformations due to the introduced bending moment that reduces the deformations in one bridge and increases them in the other. As the load is always applied in the same way and in the same position, these variations occur systematically in bridges 1 and 2 in the same way. However, the results obtained corroborate that this error, due to limitations in the load application methodology, does not have a significant effect on the results, as the errors made in the estimation of the calibration forces are less than 3.5%.

### 4.2. Results of the Device during Pedalling

In this project, the force applied to the pedal was projected in a reference system in solidarity with the crank and the pedal axis so that the axes coincide with the radial direction of the crank and the tangential force is perpendicular to it, both components being in the sagittal plane. The lateral-medial force is perpendicular to the sagittal plane and coincident with the pedal axis. This choice is similar to the one adopted by Alexander et al. [16] and different from the choice made by many authors [12,13,15,17,20,25,26], who define the reference system as solid to the pedal. This is because, usually, the sensors for measuring forces on the pedal have been placed on the pedal itself. However, with the design proposed in this work, the effective force exerted on the pedal can be obtained directly without the need for further calculation or post-processing due to the axis system used.

The results obtained are similar in value and form to those obtained by Alexander et al. [16]. Additionally, in this study the force component perpendicular to the sagittal plane was also recorded. The results obtained for this component show that this force is an order of magnitude lower than those recorded in the sagittal plane, reaching its maximum at around 90–110° of the crank angle. However, it is of interest that the maximum is always negative, i.e., it occurs laterally along the anatomical axes. This indicates that in this position the cyclist is exerting a force that has a component that moves away from the bicycle frame. When analysing the average force of the six subjects, this component shows an average value practically null during the second part of the pedalling cycle. These results are in agreement with those presented by Hull and Davis [12,13]. These authors obtained experimental measurements of pedal forces for a single participant. In the present study, forces were recorded for six different participants. Although the average lateral-medial force is similar to that presented by Hull and Davis, the temporal evolution of this force for each participant presents a great variability, being in some cases positive and in others negative. However, the curves reveal that the maximum values in this second phase of the cycle obtained in each case are very small.

From a biomechanical perspective, force peaks in this direction are important because of their influence on joint forces and moments. Although, under normal pedalling conditions, the risk of injury is low, one of the objectives of this work was to provide a tool to accurately estimate a component of force that is important in the biomechanical analysis of the cyclist. In this sense, altering the parameters of the bicycle (saddle height, Q factor) or the pedalling conditions (performing an incorrect technical gesture) can lead to overloading the joints. In these cases, the lateral-medial component of the force can play a very important role, especially in the knee, where these force values lead to abduction–adduction moments [27,28] that can cause overloads and subsequent joint injuries.

When the effective force and power generated during pedalling is analysed, both their temporal evolution and their values are in agreement with those published in the literature [16,25,26]. In particular, the maximum of the effective force and power generated occurs at a crank angle equal to 90°. At this point, the value of the effective force is very close to the value of the result of the total force because the radial force is close to zero and the axial force component is very small. This fact suggests that the instantaneous effectiveness index at this point is practically 1. Additionally, the results obtained show that during the second half of the pedalling cycle the effective force is negative, as is the pedalling power. This result is in agreement with the study published by Hull [12,13], which indicates that for crank angles greater than 180° it is the bicycle that is providing energy to the rider to help lift the foot. The results show that the negative power is slightly higher than that shown in the literature. Anyway, the net power output during one cycle ranged between 69 and 78 watts for all subjects. This range contains the value of 75 watts, which is half the power set in the test conditions for the two legs. The differences observed with respect to the ideal value could be due to asymmetries in the behaviour of the right and left legs.

In relation to the analysis of the local effectiveness index, the results obtained are similar to those shown by Hull [12,13] and Sanderson [25,26]. Thus, the analysis of the local effectiveness index can be applied as a tool for improving pedalling technique in order to maximise cyclist performance. Along the same lines, the consideration of the 3D component of pedalling force has been shown not to be relevant since the lateral-medial force component accounts for less than 1% in the calculation of the overall effectiveness index. However, this 3D component can be critical in a biomechanical analysis of the cyclist, as previously mentioned.

Analysing the pedalling performance from the effectiveness index, the longer the effective force is positive, the higher the pedalling performance will be. However, this consideration is purely mechanical and limited to the analysis of a single pedal. To rigorously consider performance analysis, the efficiency index of both pedals would have to be taken into account. This analysis is proposed for future work. Additionally, performance analysis should be considered from a biomechanical point of view. As mentioned above, from a strictly mechanical point of view, the longer the effective force is generated, the higher the performance will be. However, from a biomechanical point of view, the fatigue of the muscles involved in the pedalling task must be considered. Thus, for long pedalling intervals, the definition of a pedalling profile in which the power generated is approximately constant and the result of the sum of the power generated in both pedals in a synchronised manner would be more efficient. Thus, during the first part of the pedalling cycle, one leg would be providing power and during the second phase of the cycle, the other leg would be providing power. The consideration of biomechanical aspects in the analysis of performance is proposed for future work.

## 5. Conclusions

In the present work, a device was developed to experimentally measure 3D pedalling forces in laboratory and outdoor conditions. The main characteristics of the device are four. Firstly, as mentioned above, it is a device that can measure 3D pedal forces in outdoor conditions, which allows recording pedal force patterns in real conditions. Secondly, this device is applicable to any commercial metal-type pedal with the area where the axis is assembled to the crank accessible to the placement of gauges. Thirdly, given the characteristics of the device and the way it is mounted on the inside of the crank, it does not alter the pedalling of the cyclist. Finally, the presented device is able to register the component in the direction of the pedal axis. The results obtained in this research show that this component is an order of magnitude smaller than the forces contained in the sagittal plane. Therefore, its influence on the calculation of the effectiveness index is negligible. However, previous studies carried out by other authors show that its consideration is relevant from a biomechanical point of view due to its influence on joint forces and moments, especially in the knee.

The calibration of the device has made it possible to obtain a very accurate measurement system with errors of less than 3.5%. The implementation of gauges in a half-bridge configuration has made it possible to obtain a robust device with a high repeatability rate in the results.

The device presented allows an analysis of pedalling performance based on a local and global effectiveness index. However, this analysis has been limited because only one pedal was instrumented. In order to carry out an exhaustive analysis of the global pedalling performance of a cyclist, efficiency rates on the two pedals and biomechanical considerations such as fatigue of the muscles involved in pedalling would have to be taken into account.

## Figures and Tables

**Table 1:** Microdeformations measured by the gauges when applying a force of 98.1 N in each direction.

Wheastone Bridge	Gauge	Load Application Direction		
		T	R	LM
1	G1	20.142	−1.145	−5.230
1	G2	−21.371	1.098	−5.182
2	G3	2.779	20.764	−5.526
2	G4	3.131	−19.987	−5.202
3	G5	1.005	−76.543	67.202
3	G6	0.961	76.674	−65.305

**Table 2:** Components of the **M** matrix for different calibration loads.

Load (N)	M1 (49.05)	M2 (73.57)	M3 (98.16)	M4 (127.53)	M5 (147.15)	M6 (196.2)	M7 (294.3)
m_11_	1.520	1.542	1.526	1.499	1.499	1.558	1.558
m_12_	0.386	0.402	0.425	0.361	0.375	0.407	0.407
m_13_	0.381	0.403	0.376	0.349	0.357	0.379	0.379
m_21_	−0.033	−0.059	−0.065	−0.085	−0.095	−0.118	−0.118
m_22_	−1.651	−1.644	−1.588	−1.625	−1.651	−1.654	−1.654
m_23_	−0.131	−0.143	−0.082	−0.169	−0.155	−0.153	−0.153
m_31_	0.013	0.015	0.019	0.016	0.015	0.008	0.008
m_32_	−0.999	−0.973	−0.956	−0.958	−0.960	−0.967	−0.967
m_33_	−1.157	−1.152	−1.129	−1.156	−1.147	−1.156	−1.156

**Table 3:** Components of the **M_mean._**.

Components	M_mean_
m_11_	1.529
m_12_	0.386
m_13_	0.365
m_21_	−0.087
m_22_	−1.652
m_23_	−0.159
m_31_	0.013
m_32_	−0.971
m_33_	−1.155

**Table 4:** NRMSE of **M_mean_** (results expressed in %).

Error	Value (%)
NRMSEM_T_	2.188
NRMSEM_R_	3.398
NRMSEM_LM_	1.132
NRMSEM_GLOBAL_	2.243

**Table 5:** NRMSE of **M_mean_** (results expressed in %).

Test Direction	NRMSEM_T_ (%)	NRMSEM_R_ (%)	NRMSEM_LM_ (%)	NRMSEM_GLOBAL_ (%)
Tangential	0.118	2.782	0.783	1.229
Radial	1.117	0.250	1.278	0.884
Lateral-medial	0.331	2.849	1.418	1.529

**Table 6:** NRMSE of F_T_, F_R_ and F_LM_ (results expressed in %).

Crank Angle (°)	NRMSEM_T_ (%)	NRMSEM_R_ (%)	NRMSEM_LM_ (%)
0	1.126	0.254	1.278
45	2.097	2.556	1.340
90	0.116	2.781	0.785
135	1.010	0.846	0.604
180	0.848	0.337	0.377
225	1.668	2.634	0.950
270	1.439	4.205	0.561
315	3.276	0.744	0.910

**Table 7:** Differences and relative error between EIE_2D_ and EIE_3D_.

Participant	EIE Max ^1^		EIE Min ^2^		Error EIE Max (%)	Error EIE Min (%)
	2D	3D	2D	3D		
1	0.993	0.991	−0.992	−0.989	0.202	0.303
2	0.994	0.987	−0.994	−0.993	0.689	0.111
3	0.979	0.969	−0.988	−0.986	1.063	0.193
4	0.985	0.982	−0.983	−0.980	0.346	0.286
5	0.996	0.995	−0.990	−0.988	0.131	0.223
6	0.992	0.991	−0.986	−0.983	0.101	0.305
Mean	0.974	0.969	−0.970	−0.968	0.495	0.165

^1^ EIE maximum value. ^2^ EIE minimum value.

**Table 8:** Differences and relative error between EI_2D_ and EI_3D_.

Participant	EI		Error (%)
	2D	3D	
1	0.293	0.292	0.319
2	0.210	0.209	0.303
3	0.245	0.243	0.707
4	0.212	0.211	0.374
5	0.425	0.424	0.203
6	0.429	0.429	0.067
Mean	0.290	0.290	0.235

## Data Availability

The data presented in this study are available on request from the corresponding author. Data are not publicly available due to ethical reasons.
